# Impaired pendulum-like mechanics during post-stroke walking: a biomechanical comparison with healthy individuals

**DOI:** 10.3389/fneur.2026.1807498

**Published:** 2026-05-22

**Authors:** Serena Cerfoglio, Luca Vismara, Lorenzo Priano, Alessandro Mauro, Matteo Bigoni, Manuela Galli, Veronica Cimolin

**Affiliations:** 1 Department of Electronics, Information and Bioengineering, Politecnico di Milano, Milan, Italy; 2 Division of Neurology and Neurorehabilitation, IRCCS Istituto Auxologico Italiano, Verbania, Italy; 3 Department of Neurosciences, University of Turin, Turin, Italy

**Keywords:** energy recovery, gait biomechanics, mechanical efficiency, motion capture, stroke

## Abstract

**Introduction:**

After stroke, many individuals recover the ability to walk independently. However, post-stroke gait remains inefficient and energetically demanding. Alterations in the pendulum-like exchange of mechanical energy of the center of mass (CoM) may contribute to gait inefficiency.

**Methods:**

30 post-stroke individuals and 30 healthy controls walked at self-selected speed while 3D motion capture data were collected with an optoelectronic system. Spatio-temporal gait parameters were computed, and CoM mechanical energy was estimated from pelvic marker trajectories. Mechanical efficiency was quantified using the Energy Recovery Index (ERI) and Congruity (C) between potential and kinetic energy. Between-group differences were assessed using independent-samples *t*-tests, and analyses accounting for walking speed were performed for mechanical outcomes. Within the stroke group, correlations were computed between ERI and selected spatio-temporal and functional parameters, including the Locomotor Rehabilitation Index (LRI).

**Results:**

Compared with healthy controls, post-stroke participants exhibited significantly reduced walking speed, shorter step and stride lengths, and prolonged double support time (*p* < 0.05). Mechanical energy recovery was significantly lower in the stroke group (ERI: 54.4 ± 12.7%) than in healthy participants (68.1 ± 4.6%), while C was significantly higher (*p* < 0.05), indicating altered pendulum-like mechanics. Differences in ERI and congruity could not be explained by walking speed alone. In the stroke group, ERI was positively correlated with step length and LRI, and negatively correlated with double support time and non-plegic limb foot-off timing (*p* < 0.01).

**Discussion:**

Post-stroke walking is characterized by impaired pendulum-like mechanics of the CoM that are not solely attributable to reduced walking speed. Mechanical energy recovery is closely associated with spatio-temporal and functional gait characteristics, supporting the use of biomechanical indices such as ERI to complement conventional gait assessment and plan targeted rehabilitation strategies.

## Introduction

Stroke is a leading cause of long-term disability worldwide and it frequently results in persistent motor impairments that limit functional independence and participation in daily life (1, 2). Among these impairments, walking dysfunction represents one of the most common and disabling consequences of stroke (3, 4). Although post-stroke individuals can recover the ability to walk independently, their gait is often incomplete and characterized by alterations, such as foot drop during swing and compensatory trunk and pelvic movements (5, 6). Consequently, walking frequently remains energetically demanding, leading to reduced endurance and early fatigue. This indicates that the recovery of independent walking does not necessarily correspond to the restoration of an efficient gait pattern (7–9).

In human locomotion, efficiency is related to the ability to minimize energy expenditure for a given task. In healthy individuals, walking is remarkably efficient thanks to the coordinated interaction of neural control, musculoskeletal mechanics, and biomechanical constraints, allowing long-distance locomotion with a relatively low metabolic cost (10, 11). From a biomechanical perspective, this efficiency is closely related to the mechanical organization of movement.

During healthy walking, the body's center of mass (CoM) follows a characteristic trajectory that allows a partial exchange between gravitational potential energy and kinetic energy (12, 13). This mechanism, described by the inverted pendulum model, reduces the amount of positive mechanical work that must be actively generated by the muscles, particularly at preferred walking speeds (14, 15). Through this mechanism, mechanical energy is partially conserved across the gait cycle, contributing to reduced muscular effort. However, when this mechanical organization is disrupted, walking efficiency drops and the metabolic cost of locomotion rises (16, 17). In post-stroke populations, such inefficiency can represent a major functional limitation, sometimes exceeding the impact of isolated impairments such as weakness or spasticity (18). Consistent with this, studies have reported an increased energy cost of walking after stroke, which persists even in those who have regained independent ambulation (19, 20).

This inefficiency is closely related to the nature of post-stroke gait impairments, which extend beyond muscle weakness. Impaired selective motor activation, abnormal muscle synergies, and altered inter-segmental timing contribute to a gait pattern that relies on compensatory strategies to maintain forward progression. While these adaptations may facilitate functional ambulation, they significantly increase the mechanical work required for progression, particularly during prolonged walking tasks (21). Consequently, post-stroke gait is typically characterized by reduced walking speed, temporal and spatial asymmetries, and altered joint coordination (22). These alterations directly impact the normal pendulum-like mechanism, leading to a persistent increase in the cost of walking compared to healthy individuals, even when walking at similar or slower speeds (17, 23). This energetic demand is directly linked to increased fatigue and reduced participation in daily life also into the chronic phase after stroke, suggesting that conventional rehabilitation strategies do not always restore an energetically optimal gait pattern (19, 24).

Gait assessment in post-stroke populations has largely focused on spatio-temporal parameters and lower limb kinematics during walking, whereas fewer studies have examined the mechanical organization of gait and its contribution to efficiency (3, 25). From a biomechanical perspective, this can be quantified using indices derived from the analysis of CoM (26). In particular, the Energy Recovery Index (ERI) represents a consolidated measure of the effectiveness of pendulum-like energy exchange (13).

ERI serves a global descriptor of gait coordination rather than a simple energetic surrogate and has been extensively used to characterize mechanical efficiency in healthy walking, aging, and neurological disorders such as Parkinson's disease and cerebral palsy. For instance, reduced energy recovery has been observed in elderly individuals, reflecting age-related alterations in gait coordination and mechanical efficiency (27, 28). Similarly, studies in cerebral palsy and Parkinson's disease have highlighted alterations in the coordination of kinetic and potential energy fluctuations, resulting in impaired mechanical energy exchange and increased energetic demand (17, 29).

Despite its theoretical foundation and widespread application in biomechanics, the use of ERI in post-stroke populations remains relatively limited. While the increased cost of walking after stroke is well established (19, 20), most investigations have emphasized metabolic measures or segmental kinematics rather than the effectiveness of pendulum-like energy exchange. Specifically, it has not been fully elucidated whether the reduced walking efficiency observed after stroke primarily reflects slower locomotion (30) or a fundamental breakdown of pendulum-like mechanics that exist independently of speed. Investigating these aspects in this population may therefore offer novel insights into the mechanical organization of post-stroke gait and its clinical correlates.

The aim of this study was two-fold. First, it aimed to evaluate walking efficiency in post-stroke individuals by quantifying the mechanical energy exchange of the CoM during gait, in comparison with healthy individuals while specifically accounting for the effect of walking speed. Secondly, it aimed to investigate the relationship between walking efficiency and specific spatio-temporal gait parameters to identify which aspects of gait impairment most significantly impact mechanical coordination. By providing an objective and biomechanically grounded measure of energy exchange, this study aims to offer complementary information on gait dysfunction, assessing to what extent mechanical disorganization represents a distinct impairment beyond the simple effect of reduced velocity.

## Materials and methods

### Participants

This study follows a retrospective, cross-sectional design aimed at evaluating mechanical gait efficiency in a clinical population. Two cohorts of participants were enrolled on a voluntary basis. The first group consisted of post-stroke individuals (stroke group, SG) recruited among the inpatients of San Giuseppe Hospital (IRCCS Istituto Auxologico Italiano, Piancavallo, Italy), whereas the second group included healthy adults (healthy group, HG) recruited from the hospital's staff. Post-stroke participants were selected based on the following inclusion criteria: (i) age ≥18 years, (ii) mild-to-moderate hemiparesis affecting both upper and lower limbs, with National Institutes of Health Stroke Scale (NIHSS) scores ≤ 10 and Modified Rankin Scale (mRS) scores ≤ 3, respectively, (iii) ability to understand vocal cues, with a Mini-Mental State Examination (MMSE) score of at least 26, and (iv) ability to walk short distances (< 10m) without assistance. Individuals exhibiting bilateral motor impairment were excluded. Conversely, inclusion criteria for healthy individuals were the following: (i) age ≥18 years; (ii) no injuries within the past year, and (iii) absence of musculoskeletal or neurological disorders that could affect gait.

The sample size was defined by the number of participants meeting the inclusion criteria within the clinical database during the study period. This retrospective approach balanced practical constraints with the requirement for statistical sensitivity. To ensure robustness, a power analysis confirmed that the sample was sufficient to achieve a power of at least 80% (α = 0.05) to detect large effect sizes (Cohen's *d* = 0.80), consistent with previous biomechanical investigations (31).

The study protocol received approval from the internal Ethics Committee and was conducted in accordance with the ethical standards of the Institute and the Declaration of Helsinki (1964), including its most recent amendments, with the option to withdraw from the experimental tests at any time.

### Experimental set-up

All participants underwent 3D instrumented gait analysis (3D-GA) at the Movement Analysis Laboratory of San Giuseppe Hospital. The laboratory is equipped with a 6-camera optoelectronic motion capture (MCap) system (Vicon, Oxford Metrics, Ltd.; Oxford, UK; sampling rate: 100 Hz) and two force plates (Kistler, Winterthur, CH) embedded in the middle of a 10 m walkway.

**Figure 1:**
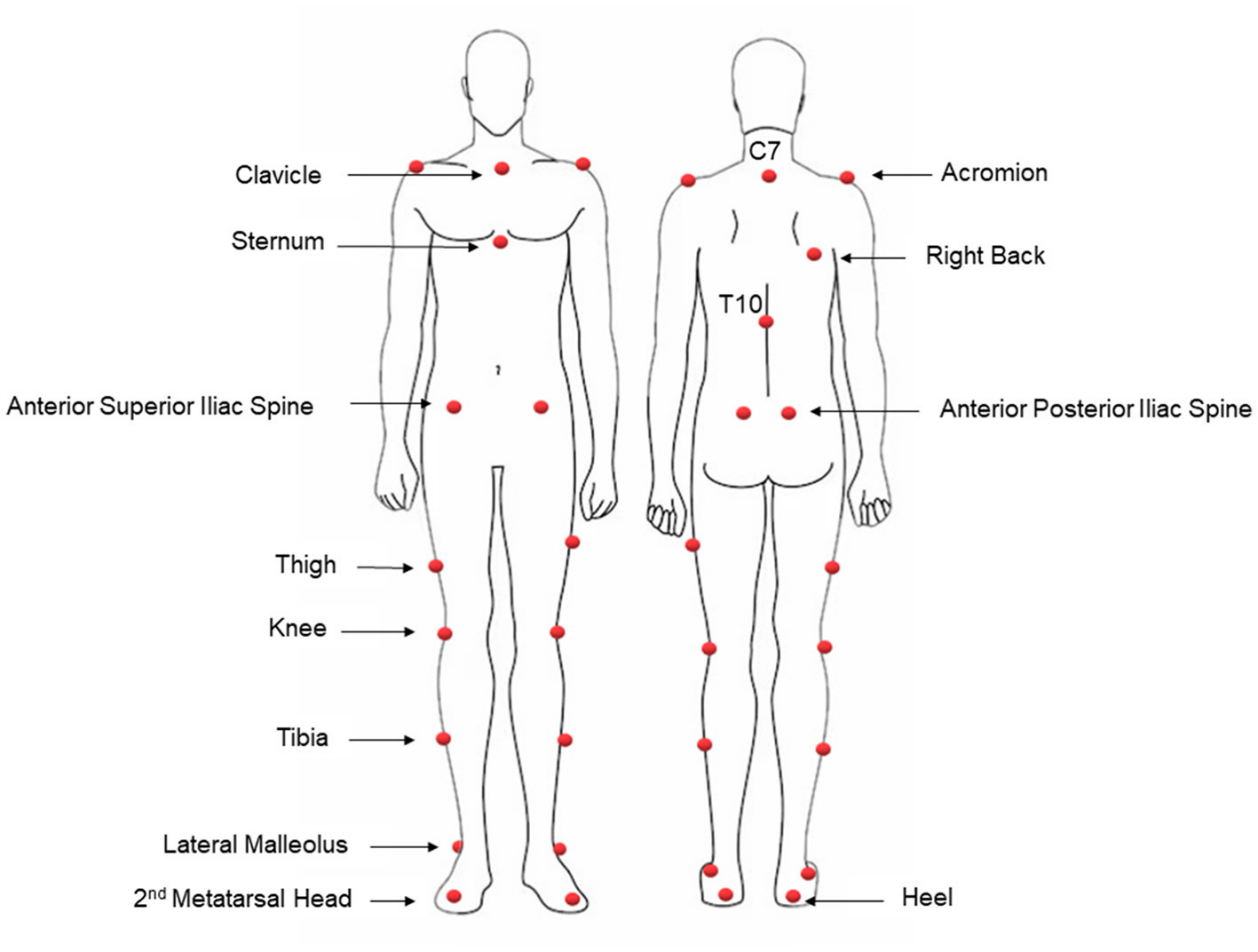
Markers' placement according to anatomical reference points of Plug-In Gait Model ((32, 33)).

Anthropometric measurements were recorded for each participant, including height, weight, distance between the anterior superior iliac spines (ASIS), leg length, and the width of knee and ankle. Subsequently, 23 spherical retro-reflective markers (Ø = 15 mm) were manually positioned on specific anatomical landmarks on participants' bodies according to Plug-In Gait model (32, 33), as displayed in Figure 1.

According to the experimental protocol and 3D-GA requirements, participants were asked to walk barefoot along the walkway at their self-selected walking speed (SSWS). Up to three successful trials were collected to guarantee the reproducibility of the results in terms of gait parameters. Trials were considered consistent if performed at a steady SSWS without interruptions.

### Data analysis and processing

Raw data collected by MCap were processed using dedicated software for data tracking (Version 1.8, VICON, Oxford Metrics Ltd., Oxford, UK) and then imported into MATLAB^®^ (Mathworks Inc, Natick, MA, USA) for further processing through *ad-hoc* and custom-written routines for computing relevant gait-related parameters.

#### Spatio-temporal parameters

Main gait events (i.e., right/left heel strike and toe off) were identified for each trial from the trajectories of the markers placed on the feet to define the gait cycle (34). Spatio-temporal parameters were then computed automatically and bilaterally using the above-mentioned dedicated software. In particular, the following parameters were considered:

- Step time (s): time interval between consecutive contralateral and ipsilateral heel strikes;
- Stride time (s): time interval between two consecutive heel strikes of the same foot;
- Step length (m): antero-posterior distance from one foot strike to the subsequent one;
- Stride length (m): distance between two consecutive heel strikes of the same foot;
- Double support (s): time in which both feet are in contact with the ground;
- Foot off (%): duration of the stance phase, as % of the gait cycle
- Cadence (steps/min): number of steps in a time unit (i.e., 1 minute)
- Walking speed or self-selected walking speed (SSWS) (m/s): ratio between stride length and stride time.

#### Mechanical parameters

According to previous studies, the body CoM was estimated from the trajectories of the markers on the pelvis, as pelvic motion provides a reliable approximation of whole-body CoM displacement during walking (35, 36). In particular, the CoM was defined as the midpoint between the vectors connecting the midpoints of the right and left anterior-superior iliac spines markers and those on the right and left posterior superior iliac spines markers (37–39). To ensure signal quality and mitigate the impact of instrumental noise, all CoM trajectories and velocities were filtered using a 4th-order zero-phase Butterworth low-pass filter with a cut-off frequency of 10 Hz.

CoM motion was computed along the anterior-posterior (AP), medio-lateral (ML) and vertical (V) directions. The potential energy (E_P_) associated with CoM displacement was computed as follows:


$$\begin{array}{l} E_{P}=mgh \end{array}$$ (1)

where m is the participant's body mass (kg), g is the gravitational acceleration (9.81 m/s^2^) and h is the vertical distance of the CoM from the ground. The total kinetic energy (E_K_) associated with CoM displacement was then computed as follows:


$$\begin{array}{l} E_{K}=\;E_{K,\;AP}+\;E_{K,\;ML}+\;E_{K,\;V}=0.5\;m\left[\left(v_{AP}^{2}+\;v_{ML}^{2}+v_{V}^{2}\right)\right] \end{array}$$ (2)

Where V_AP_, V_ML_ and V_V_ are the directional components of CoM velocities, computed as the first-order finite differences of their respective displacements. This formulation corresponds to the classical definition of E_K_ as it is based on the magnitude of the velocity vector (13). Neglecting non-vertical components would result in a systematic underestimation of total kinetic energy (40).

Finally, the total mechanical energy (E_TOT_) associated to CoM was computed as function of time (t) as the sum of potential and kinetic contributions according to the following equation:


$$\begin{array}{l} E_{TOT}\left(t\right)=E_{P}\left(t\right)+E_{K}\left(t\right) \end{array}$$ (3)

All computations were performed on a step-by-step basis, and all parameters were subsequently averaged across steps and trials to obtain a representative value for each participant. Step segmentation was performed using the previously defined gait events derived from foot marker trajectories, with each step defined as the interval between heel strike of one foot and heel strike of the contralateral foot. These events are temporally consistent with the minima of Ep, which typically occur near the step-to-step transition during double support (29, 35). To account for varying step durations across different walking speeds and to standardize the mechanical analysis, all energy-related signals for each step were time-normalized from 0 to 100% of the step cycle. Across the participants, the mean number of analyzed steps was 12.4 ± 2.02 for HG and 17.6 ± 3.22 for SG. All energy-related parameters were normalized to the participant's body mass to account for individual differences in body weight.

Over the step period, the work associated with potential energy (W_P_), kinetic energy (W_K_), and the total mechanical energy (W_TOT_) of CoM was computed as the sum of their positive increments. Finally, ERI was computed as follows:


$$\begin{array}{l} ERI=\;\frac{\left(W_{P}+\;W_{K}\right)\;-\;W_{TOT}}{\left(W_{P}+\;W_{K}\;\right)} \end{array}$$ (4)

**Figure 2:**
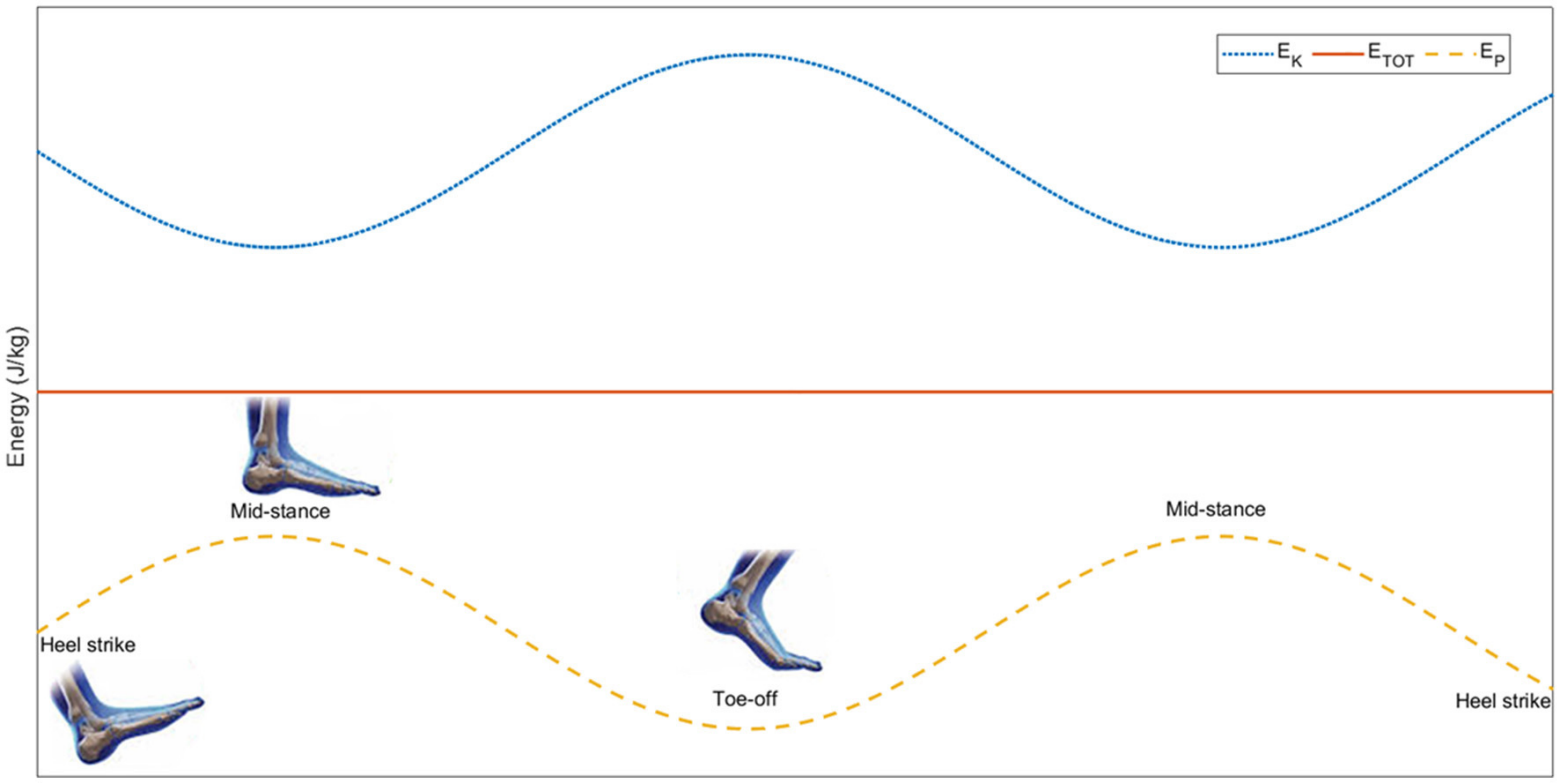
Theoretical pendulum-like exchange of CoM mechanical energy during walking. E_K_ and E_P_ vary out of phase, E_TOT_ remains constant, reflecting the behaviour of a perfectly inverted pendulum. Representative gait events (i.e., heel strike, mid-stance, and toe-off) are shown schematically to illustrate their temporal relationship with the energy fluctuations.

ERI provides a quantitative measure of the mechanical energy exchange between potential and kinetic energy of CoM within each step, following the formulation originally proposed by Cavagna et al. (13). Specifically, it represents the proportion of the combined fluctuations in potential and kinetic energy that is recovered through their out-of-phase behaviour. Figure 2 illustrates the theoretical time-course of pendular exchange between E_P_ and E_K_. In the case of a perfectly inverted pendulum, this exchange would be complete, yielding to an ERI of 100%. However, in human walking this condition is never achieved since this model represents an idealized condition. In real walking, the effectiveness of ERI depends on the relationship between E_P_ and E_K_, their relative amplitudes, and the timing of step-to-step transitions, all of which vary with walking speed (41, 42).

In healthy adults walking at SSWS, ERI values typically range between 60%−70%, reflecting an efficient pendulum-like exchange of mechanical energy (13, 26). Lower ERI values indicate a reduced effectiveness of this exchange and are commonly observed when gait mechanics are altered, such as at very slow or fast walking speeds or in pathologically altered conditions (19, 29). Accordingly, reduced ERI values (< 60%) reflect a less effective exchange, resulting in greater mechanical demand and necessitating increased active muscle work to redirect the CoM. This shift from passive dynamics to active muscular compensation ultimately explains the increased energetic cost for a given walking task.

In addition, congruity (C) was calculated to further describe the phase relationship between E_P_ and E_K_ of CoM during each step according to the formulation proposed by Sparling et al. (43):


$$\begin{array}{l} C=\;\frac{d}{dt}E_{P}\times \frac{d}{dt}E_{K} \end{array}$$ (5)

Where $\frac{d}{dt}E_{P}$ and $\frac{d}{dt}E_{K}$ represent the instantaneous rates of change of potential and kinetic energy, respectively. It quantifies the temporal correspondence between changes in E_P_ and E_K_ over the step. Therefore, higher congruity values indicate a more in-phase relationship between the two energy curves, which is typically associated with reduced energy recovery, whereas lower congruity values reflect a more out-of-phase behaviour and a greater potential for pendulum-like energy exchange. An increased congruity between potential and kinetic energy fluctuations may reflect a temporally rigid gait strategy, characterized by a reduced physiological alternation between phases of energy exchange. From a clinical perspective, this pattern is consistent with a safety-driven locomotor strategy, in which movement timing is constrained to enhance stability at the expense of mechanical efficiency (44).

#### Locomotor rehabilitation index

In addition to mechanical energy measures, the Locomotor Rehabilitation Index (LRI) was computed to account for the influence of walking speed in the evaluation of gait efficiency. Walking speed is known to influence the effectiveness of the pendular exchange between potential and kinetic energy of the CoM and, consequently, energy recovery (26, 45). LRI was therefore included to provide a normalized indicator of how closely each participant's self-selected walking speed approached a theoretically optimal walking speed (OWS).

OWS was estimated from individual anthropometric parameters according to inverted pendulum mechanics, following the approach proposed by Peyré-Tartaruga et al. (46), and it was defined as:


$$\begin{array}{l} OWS=\;\sqrt{Fr\times g\times L} \end{array}$$ (6)

Where Fr is the Froude number (47), g is gravitational acceleration, and L is the participant's leg length. LRI was then computed as the ratio between the SSWS derived from MCap trials and OWS:


$$\begin{array}{l} LRI=\;\frac{SSWS}{OWS}\times 100 \end{array}$$ (7)

While energy recovery quantifies the efficiency of mechanical energy exchange during walking, LRI reflects the selection of walking speed relative to an energetically favourable condition. The combined assessment of energy recovery and LRI allows mechanical energy exchange to be interpreted in relation to speed selection, accounting for individual differences in anthropometry.

### Statistical analysis

All statistical analyses were performed in Matlab (Mathworks Inc, Natick, MA, USA). Data distribution was assessed using the Kolmogorov–Smirnov test, and homogeneity of variances was verified using Levene's test. As the data met the assumptions of normality and homoscedasticity, variables are reported as mean ± standard deviation.

A preliminary paired *t*-test was performed to compare gait parameters between the plegic and non-plegic limbs in SG. In the absence of significant side-to-side differences, data were pooled across limbs. Then, between-group comparisons of demographic, spatio-temporal and mechanical parameters were conducted using independent-samples *t*-tests to assess differences between the healthy group (HG) and the stroke group (SG). Effect sizes (Cohen's d) were computed to quantify the magnitude of between-group differences and interpreted according to Cohen's criteria as small (*d* ≤ 0.2), moderate (0.2 < *d* < 0.8), large (0.8 ≤ *d* < 1.3), and very large (*d* ≥ 1.3) (48). In case of significant between-group differences in demographic variables potentially influencing energy recovery (e.g., age) (49), additional analyses were conducted to account for their effects by including the relevant variables as covariates in subsequent models.

Given the influence of walking speed on mechanical energy exchange and related metrics, analyses of covariance (ANCOVA) were conducted for ERI and C, with group as a fixed factor and SSWS included as a continuous covariate. This approach allowed evaluation of group-related differences in mechanical outcomes while accounting for the effect of gait velocity and its interaction with group.

Finally, correlation analyses were performed within SG to further characterize the relationship between mechanical efficiency and gait characteristics. In particular, Pearson's correlation (*r*) coefficients were computed to examine the associations between the ERI and a selected sub-set of spatio-temporal gait parameters, including step length, double support time, foot-off, and LRI. Statistical significance was set at *p* < 0.05 for all analyses.

## Results

Participant characteristics are summarized in Table 1. Following the selection procedure described in the methods, 30 post-stroke (SG) and 30 healthy (HG) participants were included in the analysis. The post-stroke group was significantly older and had a higher body mass compared with the healthy group (*p* < 0.05), whereas no significant between-group differences were observed in body height.

**Table 1:** Participant demographic and anthropometric characteristics.

Variable	SG (*n*= 30)	HG (*n* = 30)
Participants (M/F)	19/11	11/18
Age (years)	63.70 ± 12.99^*^	38.30 ± 11.23
Height (m)	1.69 ± 0.10	1.70 ± 0.07
Weight (kg)	84.37 ± 12.01^*^	65.67 ± 9.59

SG, stroke group; HG, healthy group; M, male; F, female.

Values are reported as mean ± standard deviation, unless otherwise specified. Group comparisons were performed using independent-samples t-tests.

“^*^” = p < 0.05.

### Between-group comparisons

Preliminary paired *t*-test revealed significant inter-limb differences for foot-off timing and step time for the plegic limb, whereas no significant differences were observed for the other parameters in SG (all *p* > 0.05).

Spatio-temporal gait parameters are reported in Table 2, whereas mechanical and energetic parameters are summarized in Table 3. Independent samples *t*-tests revealed significant differences across all spatiotemporal, mechanical, and energetic variables (*p* < 0.05). The magnitude of these differences consistently large to very large, with effect sizes ranging from 0.80 to 3.81 in terms of Cohen's d, reflecting the high impact of the pathology on gait. Compared with healthy participants, post-stroke individuals exhibited significantly lower SSWS and cadence, as well as shorter step and stride lengths, together with prolonged double support and stride times, with d ranging from 1.59 to 3.81. In addition, post-stroke participants walked at a substantially lower proportion of their OWS, as reflected by a significantly reduced LRI (*d* = 3.73), despite comparable OWS between groups (Table 4). These spatio-temporal alterations were accompanied by significantly reduced potential (*d* = 2.09), kinetic (*d* = 3.14), and total mechanical work (*d* = 1.61) normalized to body mass, and by a lower ERI (*d* = 1.43) in SG. Conversely, congruity of the pendular exchange was significantly higher in post-stroke participants compared with healthy controls (*d* = 1.50).

**Table 2:** Spatio-temporal gait parameters in both groups.

Variable	SG (***n*** = 30)		HG (*n* = 30)
Cadence (steps/min)	83.21 ± 18.78^*^		118.9 ± 8.09
Double support (s)	0.53 ± 0.30^*^		0.19 ± 0.04
Foot off (%)	*Plegic*	61.30 ± 3.40	59.81 ± 1.39
	*Non-plegic*	71.05 ± 6.66^*^	
Step length (m)	0.40 ± 0.10^*^		0.71 ± 0.08
Stride length (m)	0.78 ± 0.21^*^		1.43 ± 0.16
SSWS (m/s)	0.56 ± 0.23^*^		1.42 ± 0.23
Stride time (s)	1.52 ± 0.36^*^		1.01 ± 0.07
Step time (s)	*Plegic*	0.85 ± 0.20^*^	0.51 ± 0.03
	*Non-plegic*	0.68 ± 0.20^*^	

SG, stroke group; HG, healthy group; SSWS, self-selected walking speed.

Values are reported as mean ± standard deviation. Plegic and non-plegic values refer to the affected and unaffected limbs in the post-stroke group.

“^*^” = p < 0.05.

**Table 3:** Descriptive statistics (mean ± standard deviation) of mechanical and energy parameters during walking in both groups.

Variable	SG (*n* = 30)	HG (*n* = 30)
W_P_ (J/kg)	0.21 ± 0.09^*^	0.43 ± 0.12
W_K_ (J/kg)	0.14 ± 0.09^*^	0.51 ± 0.14
W_TOT_ (J/kg)	0.16 ± 0.07^*^	0.30 ± 0.10
ERI (%)	54.44 ± 12.66^*^	68.08 ± 4.61
Congruity (%)	22.27 ± 8.22^*^	12.25 ± 4.72

SG, stroke group; HG, healthy group; WP, potential mechanical work; WK = kinetic mechanical work; WTOT = total mechanical work; ERI = Energy Recovery Index.

“^*^” = p < 0.05.

**Table 4:** Descriptive statistics (mean ± SD) of OWS and LRI for both groups.

Variable	SG (*n*= 30)	HG (*n*= 30)
**OWS (m/s)**	1.41 ± 0.06	1.44 ± 0.03
**LRI (%)**	40.01 ± 16.55^*^	98.20 ± 14.57

SG, stroke group; HG, healthy group; OWS, optimal walking speed; LRI, Locomotor Rehabilitation Index.

“^*^” = p < 0.05.

Given the significant between-group differences observed in age and body weight, additional ANCOVA models were performed to control their potential confounding effects on ERI. The effect of group on ERI remained significant when adjusting for age and body weight (both *p* < 0.001), whereas neither age nor body weight showed a significant association with ERI (*p* = 0.185 and *p* = 0.937, respectively).

**Figure 3:**
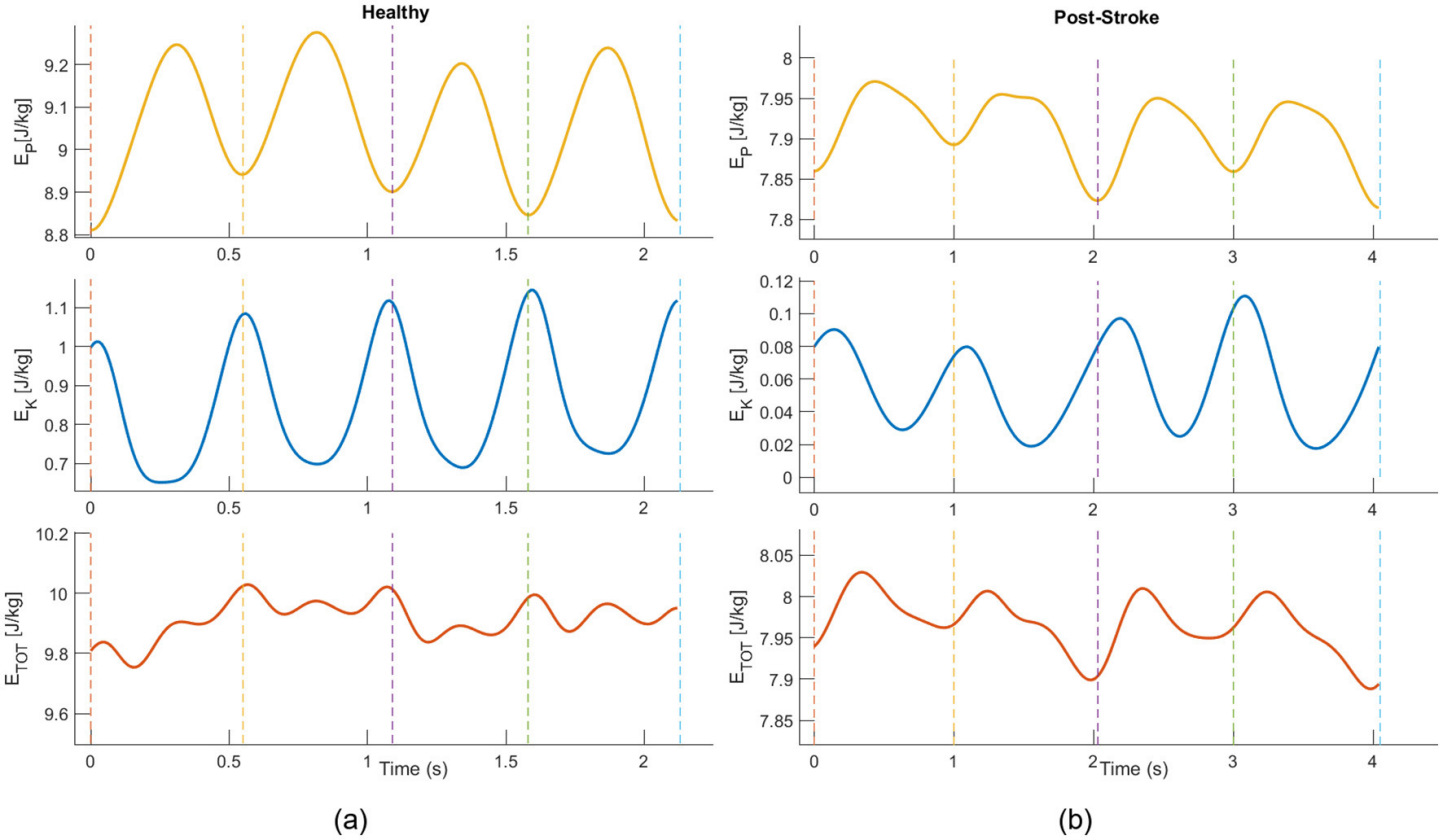
Evolution of CoM mechanical energies during walking for a representative healthy individual **(a)** and a post-stroke individual **(b)** Vertical dashed lines indicate consecutive minima of potential energy, corresponding to the step-to-step transition. Time is expressed relative to the first potential energy minimum.

Figure 3 illustrates representative profiles of the center-of-mass kinetic and potential energy across consecutive steps for a healthy participant and a post-stroke participant. In the healthy participant, kinetic and potential energy exhibit a predominantly out-of-phase pattern over time, consistent with an effective pendulum-like exchange. In contrast, in the post-stroke participant, the temporal coordination between kinetic and potential energy is altered, with increased overlap between the two components and a reduced alternation across successive steps.

### Effects of walking speed on mechanical outcomes

Given the significant between-group differences in SSWS observed in the t-tests, additional analyses were conducted to determine whether group differences in mechanical outcomes, specifically ERI and C, were attributable solely to reduced gait velocity. For both outcomes, ANCOVA revealed significant effects of walking speed, together with significant Group × SSWS interactions (ERI: F(1,56) = 22.29, *p* < 0.001; C: F(1,56) = 8.26, *p* = 0.006), indicating that the relationship between walking speed and mechanical outcomes differed between healthy and post-stroke participants. In addition, significant group effects were observed for both ERI (F(1,56) = 16.22, *p* < 0.001) and C (F(1,56) = 5.01, *p* = 0.029). Overall, these findings indicate that mechanical impairments in post-stroke gait are not explained solely by reduced walking speed, but are likely associated with pathology-related alterations in gait efficiency and coordination.

### Correlation between mechanical energy recovery and gait parameters

**Figure 4:**
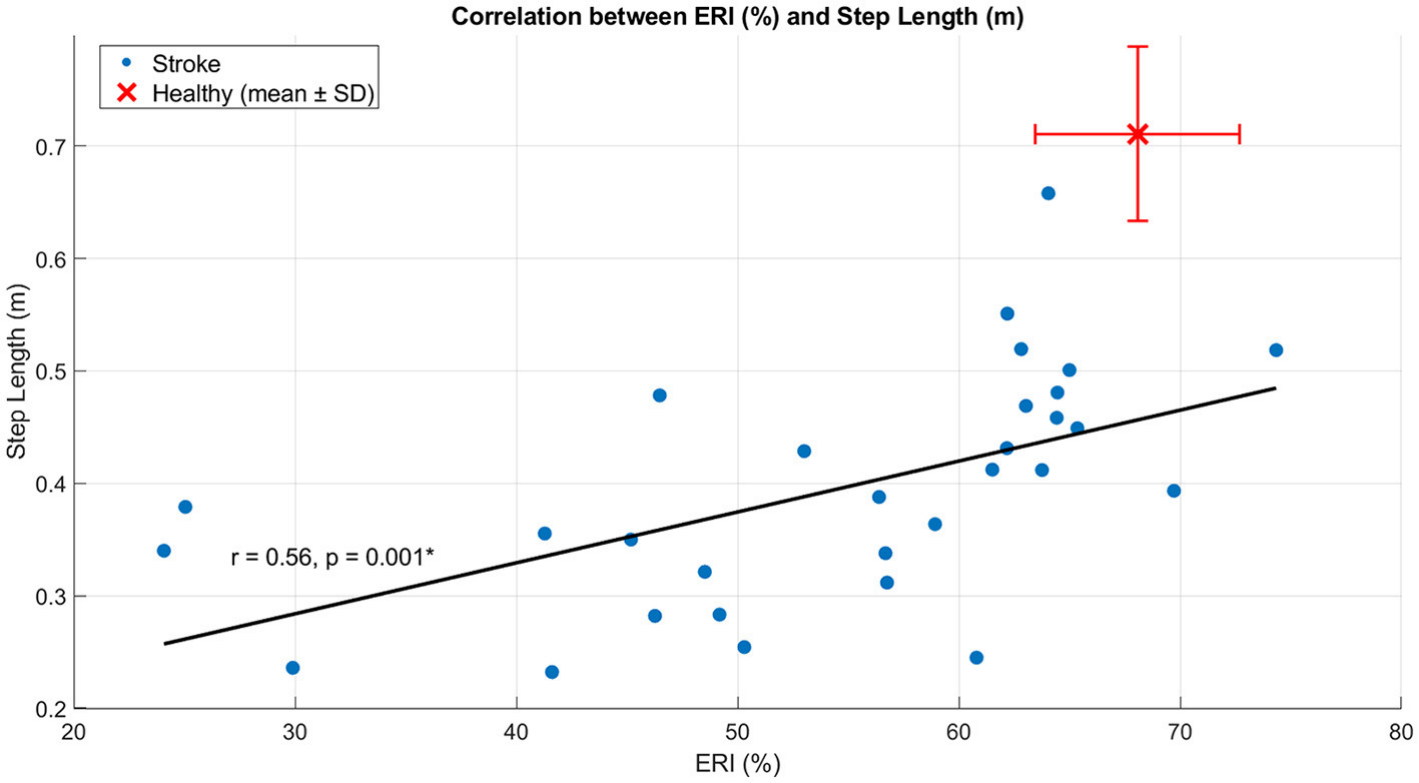
Scatter plot showing the correlation between ERI and step length in SG. Blue dots represent individual participants, the solid line indicates the linear regression fit, and the red marker denotes the mean ± SD of the healthy group. Pearson's r and the corresponding *p*-value are reported in the panel. *p < 0.05.

**Figure 5:**
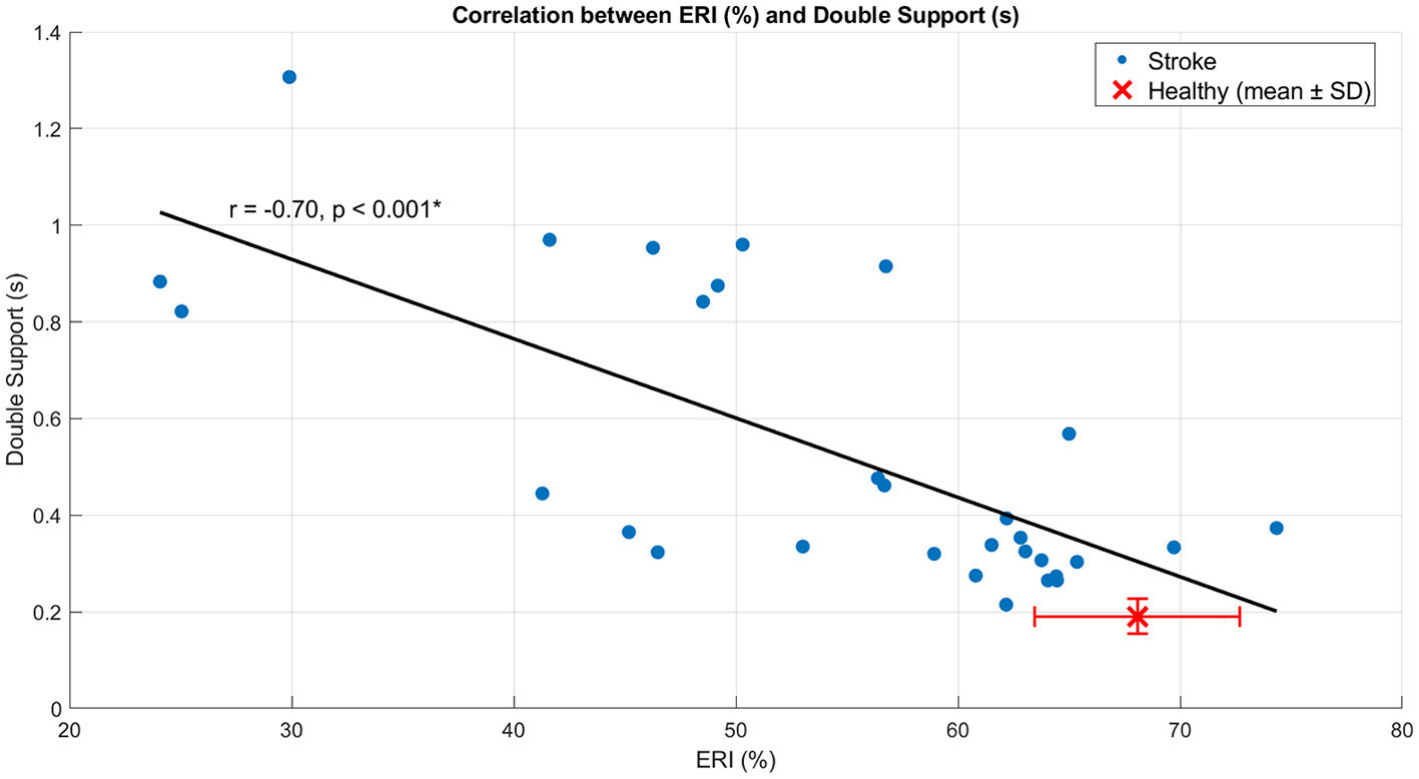
Scatter plot showing the correlation between ERI and double support in SG. Blue dots represent individual participants, the solid line indicates the linear regression fit, and the red marker denotes the mean ± standard deviation of the healthy group. Pearson's r and the corresponding *p*-value are reported in the panel. *p < 0.05.

**Figure 6:**
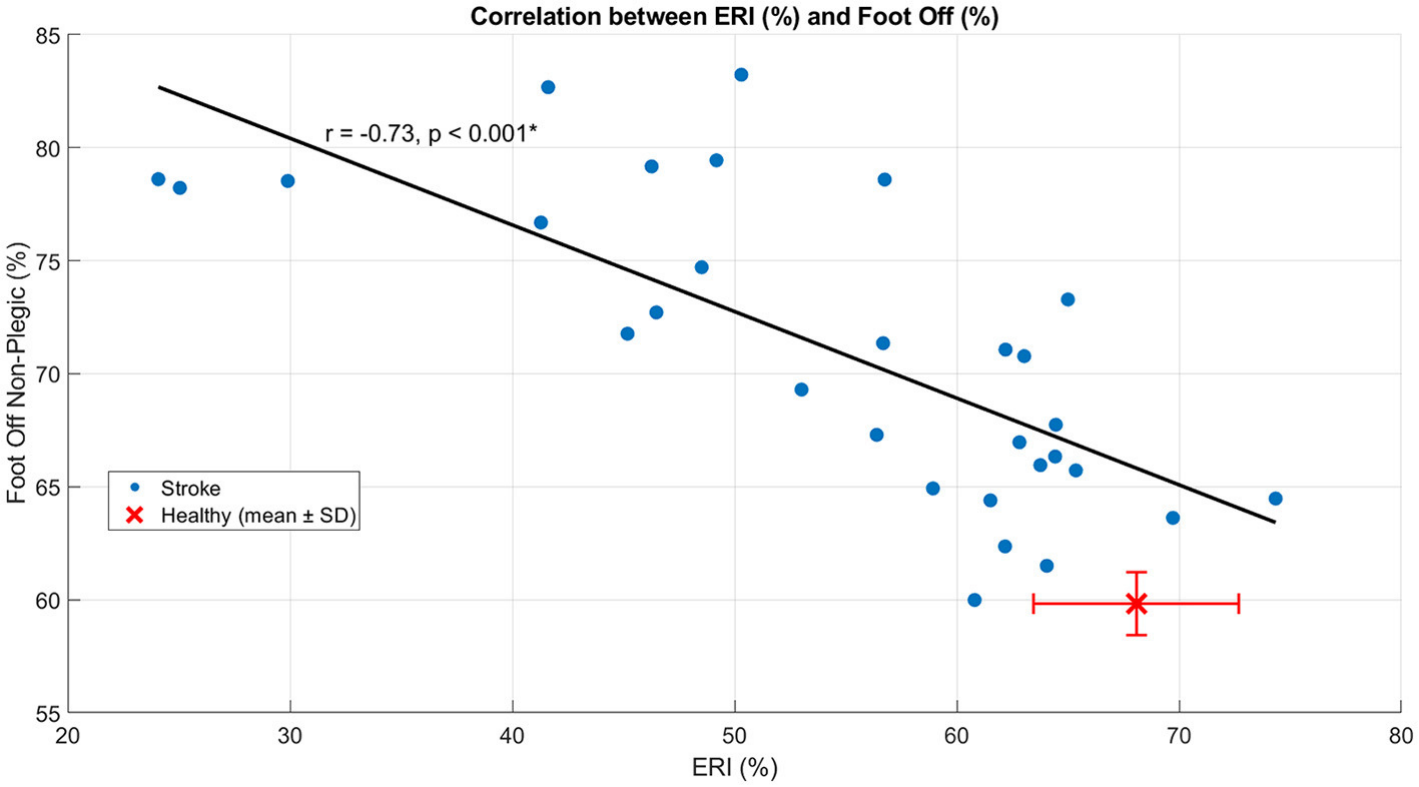
Scatter plot showing the correlation between ERI and foot off in SG. Blue dots represent individual participants, the solid line indicates the linear regression fit, and the red marker denotes the mean ± SD of the healthy group. Pearson's r and the corresponding *p*-value are reported in the panel. *p < 0.05.

**Figure 7:**
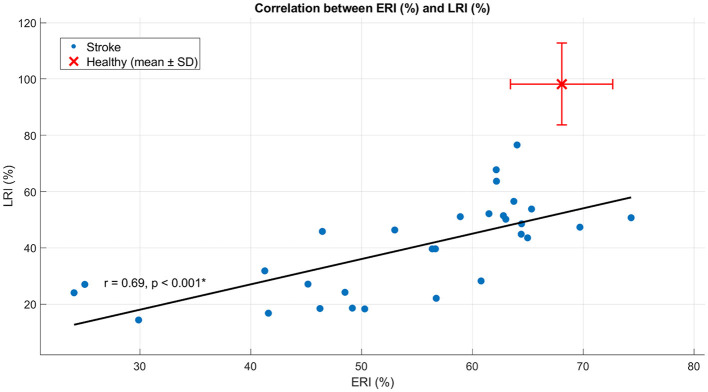
Scatter plot showing the correlation between ERI and LRI in SG. Blue dots represent individual participants, the solid line indicates the linear regression fit, and the red marker denotes the mean ± SD of the healthy group. Pearson's r and the corresponding *p*-value are reported in the panel. *p < 0.05.

In SG, ERI showed a moderate positive correlation with step length (*r* = 0.55, *p* = 0.002) (Figure 4), indicating that higher energy recovery was associated with longer steps. In contrast, ERI was strongly and negatively correlated with double support time (*r* = −0.70, *p* < 0.001) (Figure 5), indicating that lower energy recovery was associated with prolonged double support. A strong negative correlation was also observed between ERI and non-plegic limb foot-off timing (*r* = −0.73, *p* < 0.001) (Figure 6), indicating that delayed foot-off of the non-plegic limb was associated with reduced global energy recovery. Finally, ERI showed a strong positive correlation with LRI (*r* = 0.69, *p* < 0.001) (Figure 7), indicating that greater mechanical energy recovery was associated with higher levels of functional walking performance. Together, these findings indicate that global energy recovery is consistently associated with spatial, temporal, timing, and functional characteristics of gait in post-stroke participants.

## Discussion

The present study investigated the mechanical efficiency of walking in post-stroke individuals by analyzing the pendulum-like exchange of mechanical energy of the CoM and its relationship with gait characteristics. Compared with the HG, the SG exhibited marked alterations in spatio-temporal parameters, including reduced walking speed, shorter step and stride lengths, and prolonged double support time, in agreement with previous reports on post-stroke gait dysfunction (22, 50). These changes were accompanied by significantly reduced mechanical energy recovery and increased congruity between potential and kinetic energy, indicating an altered organization of the pendulum-like exchange described for healthy walking (12).

Spatio-temporal and mechanical parameters in the HG were consistent with literature values for self-selected walking speeds (35). The observed ERI values (ERI = 68.08 %) fell within the expected range for healthy adults, providing a robust baseline for the comparison. In contrast, the SG showed a marked reduction in recovery (ERI = 54.44 %) and increased C, indicating a shift toward a more in-phase behavior of potential and kinetic energy exchange, a hallmark of pathological gait. Biomechanically, this indicates that kinetic and potential energy are not effectively exchanged, leading to a more discontinuous progression where energy is prematurely dissipated and compensated by increased positive muscular work during step-to-step transitions. These findings are consistent with previous evidence showing impaired mechanical energy recovery at non-optimal walking speeds and in pathological conditions where the synergy between energy components is lost (29, 40).

The cost of this active work is intrinsically linked to the velocity at which the individual moves. Walking speed is a major determinant of mechanical energy exchange, with maximal recovery occurring near the energetically OWS predicted by inverted pendulum mechanics (14, 15). In line with this, the SG walked at lower self-selected speeds and exhibited a markedly reduced LRI, indicating a deviation from the theoretically optimal condition (46, 47). However, as highlighted by the ANCOVA analysis, speed alone does not explain the loss of mechanical efficiency in the SG. Even after accounting for speed, a significant deficit in energy recovery remains, suggesting a complex interaction between speed and efficiency. Neuro-mechanical constraints, such as impaired ankle push-off and spasticity, may limit the ability to benefit from increased speed. Thus, the observed alterations in ERI and C reflect pathology-related alterations in gait coordination and pendular energy exchange rather than a simple consequence of slower walking (17, 19, 21).

Correlation analyses further highlighted the functional relevance of energy recovery in post-stroke walking. Higher ERI values were associated with longer step length and reduced double support time, indicating that individuals who adopted less cautious and more dynamically efficient gait patterns were better able to exploit pendulum-like mechanics. Prolonged double support likely reflects a stability-oriented compensatory strategy that, while functionally adaptive, limits the effectiveness of mechanical energy exchange (22). Notably, delayed foot-off timing of the non-plegic limb was strongly associated with reduced recovery, suggesting that global mechanical inefficiency may arise not only from impairments of the plegic limb, but also from adaptive timing strategies of the non-plegic limb that shape the overall temporal organization of the gait cycle (50, 51). The strong association between ERI and LRI further emphasizes the link between mechanical efficiency and functional walking performance. Individuals who walked closer to their optimal walking speed also exhibited more effective energy exchange. This relationship suggests that patients with greater residual functional capacity are better able to coordinate the pendular mechanism. In post-stroke walking, the LRI does not reflect just a velocity choice, but the global functional integrity of the gait control system. Rather than a simple effect of velocity, these results indicate that both gait organization and speed selection reflect the degree of motor recovery after stroke.

From a clinical perspective, these findings suggest that biomechanical indices such as ERI and C can provide complementary information on gait efficiency beyond traditional measures. By characterizing the quality of energy exchange, they may help identify inefficient gait strategies that are not apparent from spatio-temporal parameters alone. Reduced ERI may reflect specific motor control impairments that can be directly targeted during interventions. In particular, low ERI indicates impaired step-to-step transitions and inefficient redirection of the CoM, suggesting the need to improve push-off effectiveness and dynamic weight transfer.

Moreover, the associations between ERI and spatio-temporal parameters can provide further clinically relevant insights. For instance, the relationship with double support suggests that reducing prolonged double support (e.g., through balance or perturbation-based training) may improve mechanical efficiency, while the correlation with step length indicates that promoting longer and more symmetric steps may enhance pendulum-like energy exchange. Accordingly, rehabilitation programs could incorporate gait training at progressively increasing speeds to approach optimal walking speed, exercises targeting ankle plantarflexor function to improve push-off, and inter-limb coordination training to optimize gait timing. These indices may also be used to objectively monitor the effects of rehabilitative interventions or assistive devices, such as Ankle-Foot Orthosis (AFO), by assessing their impact on compensatory effort and energy dissipation during walking.

In this context, ERI and congruity may therefore serve as practical biomarkers to evaluate the quality of motor recovery and the energetic sustainability of the gait pattern during clinical follow-up. Interventions should not only aim to restore independent walking or OWS, but also the out-of-phase pendular mechanism, thereby reducing the physical effort required for community ambulation and social participation. Increased energetic cost is a major limitation to walking endurance after stroke (18, 24), and interventions targeting coordination and CoM dynamics have been shown to reduce the energetic demands of post-stroke walking, supporting the clinical relevance of mechanical efficiency measures (52).

Some limitations should be acknowledged. The estimation of the CoM from pelvic markers is a well-established approach in gait biomechanics and has been widely adopted in the literature (35, 37). However, it remains an approximation of whole-body motion. Specifically, in post-stroke individuals, compensatory trunk and arm dynamics may also introduce potential confounding effects on the CoM trajectory that this simplified model might not fully capture, potentially impacting the absolute values of mechanical energy exchange. Nevertheless, this approach is sensitive enough to detect macro-biomechanical changes in pendular energy recovery. Moreover, the retrospective and cross-sectional nature of the study did not allow for evaluation of gait mechanics across systematically controlled walking speeds, nor causal inferences regarding the effects of rehabilitation on mechanical efficiency. Future studies should therefore investigate longitudinal changes in energy recovery under different walking speed conditions and following targeted gait interventions, as well as include age-matched control groups. Importantly, future research should integrate these biomechanical measures with direct assessments of metabolic cost, such as oxygen consumption measurements. Combining mechanical energy analysis with respirometry would help determine the extent to which inefficient pendular exchange directly translates into increased systemic fatigue and a higher cost of transport.

## Conclusions

This study indicates that post-stroke walking is characterized by impaired pendulum-like mechanics, reflected by reduced mechanical energy recovery and altered phase relationships between potential and kinetic energy. These alterations are not solely explained by reduced walking speed and are closely associated with spatio-temporal, timing, and functional gait characteristics. While the findings should be interpreted in the light of the study's cross-sectional retrospective design and the use of a simplified pelvis-based CoM estimation, they highlight the value of biomechanical indices such as ERI in providing complementary information on gait efficiency and coordination beyond conventional gait measures. Integrating mechanical efficiency metrics into gait assessment may improve the understanding of post-stroke walking impairments and support the development of more targeted rehabilitation strategies. Specifically, these indices could assist in evaluating and tailoring interventions focused on restoring inter-limb coordination and pendular dynamics through gait-retraining or the use, for instance, of orthotic devices. Ultimately, restoring a more efficient pendulum-like energy exchange may represent a key target to reduce the energetic cost of walking and improve functional mobility in post-stroke individuals.

## Data Availability

The datasets presented in this study can be found in online repositories. The repository and accession number can be found at: https://doi.org/10.5281/zenodo.18450713.
